# Change in the Distribution of Phosphorus Fractions in Aggregates under Different Land Uses: A Case in Sanjiang Plain, Northeast China

**DOI:** 10.3390/ijerph16020212

**Published:** 2019-01-14

**Authors:** Hu Cui, Yang Ou, Lixia Wang, Baixing Yan, Lu Han, Yingxin Li

**Affiliations:** 1 Key Laboratory of Wetland Ecology and Environment, Northeast Institute of Geography and Agroecology, Chinese Academy of Sciences, Changchun 130102, China; cuihu@iga.ac.cn (H.C.); ouyang@iga.ac.cn (Y.O.); yanbx@neigae.ac.cn (B.Y.); 18845294305@163.com (L.H.); liyingxin18@mails.ucas.ac.cn (Y.L.); 2 College of resource and environment, University of Chinese Academy of Sciences, Beijing 101408, China; 3 College of Earth Sciences, Jilin University, Changchun 130061, China

**Keywords:** Sanjiang Plain, soil aggregates, phosphorus fractions, adsorption isotherm simulation

## Abstract

Phosphorus in agro-ecosystems has attracted much attention due to its impact on the nutrient supply of plants and the risk of loss of non-point source pollution. This study investigated the fraction distribution and release of phosphorus from soil aggregates structure under different land uses (rice, maize and soybean). The soil aggregates were characterized as large macro-aggregates (L-mac, >1 mm), small macro-aggregates (S-mac, 0.25–1 mm), micro-aggregates (MIC, 0.053–0.25 mm) and silt clay (SC, <0.053 mm) with the wet-sieving method. A sequential chemical extraction scheme was used to separate phosphorus into labile inorganic phosphorus (L-Pi), labile organic phosphorus (L-Po), moderately labile organic phosphorus (Ml-Po), iron-aluminum bound phosphorus (Fe.Al-P), calcium-magnesium bound phosphorus (Ca.Mg-P), humic phosphorus (Hu-P) and residual phosphorus (Re-P). Experimental results indicated that soil aggregates were mainly S-mac and MIC, followed by L-mac and SC, and they accounted for 52.16%, 25.20%, 14.23% and 8.49% in rice fields, 44.21%, 34.61%, 12.88% and 8.30% in maize fields, and 28.87%, 47.63%, 3.52% and 19.99% in soybean fields, respectively. Total nitrogen (TN), soil organic matter (SOM), Fe and Mn in soil aggregate fractions decreased with the reduction in soil aggregate grain-sizes. For phosphorus fractions (P-fractions), Fe.Al-P and Re-P tended to condense in L-mac and S-mac. MIC and SC were the primary carriers of Ca.Mg-P. Adsorption isotherm simulation results demonstrated that L-mac and S-mac have a strong capacity to retain phosphorus. In rice fields, phosphorus bioavailability and utilization rate were high. However, the P-fractions there were easily changed under aerobic-anaerobic conditions. Therefore, the risk of phosphorus loss during drainage should be given considerable attention.

## 1. Introduction

Agricultural non-point source (NPS) pollution has seriously jeopardized the quality of the aquatic eco-environment because of the excessive amount of chemical fertilizers used in agricultural production [1,2]. Data from the China Statistical Yearbook reveal that annual chemical fertilizer application in China is 2.6-fold as that of the United States and 2.5-fold as that of the European Union [3,4]. However, phosphoric fertilizer applied to farmland can form insoluble phosphates adsorbed by soil minerals or retained by organisms, thereby resulting in its quarterly utilization rate of only 10–20% [5]. Consequently, 60% of China’s underground water is unsuitable for human consumption and China’s per capita renewable freshwater is only one-third of the world’s average level [1,4]. Furthermore, the proportion of water eutrophication caused by agricultural NPS phosphorus increased from 41% in 1950 to 77% in 2016 [3]. Therefore, examining phosphorus circulation in agro-ecosystems is scientifically important for specifying optimal fertilization regime and preventing surrounding water eutrophication.

Available phosphorus fraction (P-fraction) is an important parameter for measuring future internal phosphorus loading [6]. Therefore, the long-term behavior of phosphorus in promoting the eutrophication of freshwater can be more effectively evaluated on the basis of different P-fractions than total phosphorus (TP) content [6,7]. Labile phosphorus, including labile inorganic phosphorus (L-Pi), labile organic phosphorus (L-Po) and moderately labile organic phosphorus (Ml-Po), is weakly adsorbed on sediment colloids and calcium carbonate [8,9], is a bio-available P-fraction, and is regarded as an indicator to evaluate capacity phosphorus supply to crops [10]. The redox-sensitive iron-aluminum bound phosphorus (Fe.Al-P) can be changed into a bio-available P-fraction under anoxic conditions with the reduction in Fe^3+^ to Fe^2+^ and accumulation of soil organic matter (SOM) that competed for adsorption sites [11,12,13]. Therefore, Fe.Al-P concentration can characterize soil phosphorus recharge potential. Calcium-magnesium bound phosphorus (Ca.Mg-P) is a nonvalent inorganic phosphorus (IP) component with a weak release only under acidic conditions [14,15]. Humic phosphorus (Hu-P), which is a primary component of organic phosphorus (OP), can be separated from SOM by the destruction of the metal bridge under reducing conditions [16]. Hu-P is easily lost with surface runoff due to its low fixation degree with soil particles [10]. Previous studies have demonstrated that different land uses influence the distribution and transformation of P-fractions in agro-ecosystems through root exudates [17]. However, only a few studies have been performed on the availability and release risk of soil phosphorus from the perspective of the difference in availability and response of P-fractions to environmental changes in soil aggregates with different grain-sizes.

Soil aggregates are essential building blocks of soil structure formed by repeated polymerization of organic-inorganic composite colloids, which can determine the distribution characteristics of microbial communities and nutrients, such as carbon, nitrogen and phosphorus [18,19]. Cultivated crops vary in their excreta composition and may selectively generate soil microorganisms [20,21], which are an important regulator for SOM kinetics during the process of aggregate formation [22]. Microorganisms instinctively gather soil particles to modify their habits and indirectly stimulate the formation of coarse-aggregates (>0.25 mm) [1]. However, microorganisms can also directly consume the organic binding agents that hold aggregates together; this condition promotes the formation of fine-aggregates (<0.25 mm) enriched with extracellular polymers [20]. Nevertheless, bio-geochemical behavior of P-fractions is dominated by a combination of microbial communities and SOM. Therefore, the difference in microbial communities and SOM caused by different cultivated crops will affect the distribution and transformation of P-fractions in soil aggregates.

Phosphorus availability is the focus of research on phosphorus bio-geochemical circulation in agro-ecosystems. Therefore, clarifying the mechanism of P-fractions transformation and identifying the primary environmental factors controlling P-fractions distribution are important for improving chemical fertilizer utilization and decreasing agricultural NPS phosphorus pollution. Sanjiang Plain, which is a popular commodity production base for rice, corn and soybean, is located at the junction of China and Russia. During drainage and rainstorms, a large amount of waste water derived from farmland flows into the Okhotsk Sea via Amur River, thereby posing a potential threat to ecological security of regional black soil and aquatic ecosystem. To date, despite considerable advances in our understanding of phosphorus bio-geochemical circulation in agro-ecosystems [23,24], the accurate essence of distribution and transformation of P-fractions in soil aggregates remains poorly understood [6,12,14]. Sanjiang Plain is constantly experiencing seasonal freezing-thawing, which will complicate the formation and fragmentation of soil aggregates and the bio-geochemical circulation of P-fractions. On the base of the hypothesis that different cultivated crops affect the structure and stability of soil aggregates and thus affect the retention-release mechanism of P-fractions in there, we studied the difference in the mass proportion of soil aggregates with different grain-sizes and their chemical properties. The enrichment characteristics of P-fractions were analyzed. The phosphorus availability and release risk in agro-ecosystems under different cultivated crops (rice, maize and soybean), which is the typical land use type in northeast China, were evaluated. Experimental results are expected to provide important scientific value for formulating an optimal fertilization program, protecting black soil ecological security, and preventing agricultural NPS pollution.

## 2. Materials and Methods

### 2.1. Sampling Location

Sanjiang Plain (45°01′–48°28′ N, 130°13′–135°05′ E), which is a winter-cold zone in the northeast of China, is the confluence catchment of Amur, Wusulijiang and Songhuajiang rivers. This area is located in a temperate semi-humid continental monsoon climate with mean annual temperature of 1–4 °C, mean annual precipitation of 500–650 mm, 70–85% concentrated in June to October, a frost-free period of 120–140 days, and a freezing period of 7–8 months. The proportion of cultivated land in the Sanjiang Plain increased from 7.2% in 1949 to 33.5% in 1996, thereby becoming an important commodity grain production base with a commodity rate of 70% in the country. Nevertheless, wasteland reclamation in Sanjiang Plain has caused incalculable damage to the natural ecological environment, including a sharp decline in forest, grassland and wetland area and a reduction in wild species biodiversity. Therefore, relevant government departments have taken corresponding measures to prohibit wasteland reclamation since 2000. To date, the area of cultivated land in the Sanjiang Plain has been around 300 ha, and the annual grain output has reached 15 million tons.

Nine sample quadrats (3 m × 3 m) were set up into different crop types including maize, rice and soybean. Five topsoil (0–15 cm) were collected in diagonal line and mixed into one sample in each quadrat. According to the Chinese soil classification system, the soil type is characterized as swamp soil. The maize and soybean fields are separated by a drainage ditch, which is less than 2000 m away from the paddy field. Therefore, the regional farmland is derived from the same soil background value. In addition, regional terrain is flat and can effectively prevent the disturbance of surface runoff between farmland. The chemical properties of soil samples are shown in Table 1.

### 2.2. Soil Aggregate Segregation

Soil aggregates were separated by a wet-sieving procedure as summarized by Wang et al. [25]. Operationally, soil particles were characterized as large macro-aggregate (L-mac, >1 mm), small macro-aggregate (S-mac, 0.25–1 mm), micro-aggregate (MIC, 0.053–0.25 mm) and silt clay (SC, <0.053 mm). Specifically, soil samples were air-dried at room temperature, gently smashed into clods with a 1-cm diameter along the natural structure surface of the original soil, and placed into a set of sieves with aperture of 1, 0.25 and 0.053 mm from top to bottom. Deionized water was added to submerge soil by about 2 cm, soaked for 10 min and shook at an oscillation of 50 times/min for 2 min. Subsequently, the residue was transferred to a 250-mL beaker and oven-dried (60 °C) to constant weight.

### 2.3. Phosphorus Sorption Isotherm Experiment

Adsorption followed by desorption experiments were performed in a chamber. The 50-mL centrifuge tubes constituted 1.00 g of air-dried soil aggregates with different grain-sizes (>1 mm, 0.25–1 mm, 0.053–0.25 mm and <0.053 mm) and 25 mL of 0.01 M NaCl solution containing 0, 2, 5, 10, 20 and 40 mg/L of phosphorus as KH_2_PO_4_ were shaken for 24 h with a constant temperature of 25 °C, centrifuged at an oscillation of 8000 r/min for 10 min, digested with H_2_SO_4_ and K_2_S_2_O_8_ in an autoclave (1.1 kg/cm, 120 °C, 30 min) and filtered through 0.45-µm microfiltration membrane for determination of phosphorus. For the desorption experiment, the residue was washed twice with 25 mL of NaCl solution. Then, 30 mL of 0.01 M NaCl solution was selected as extractant. The subsequent operation is the same as the adsorption test. Deliberately, 2–3 drops of chloroform (CHCl_3_) were added to prevent microbial activity during the phosphorus adsorption and desorption processes.

On the basis of the results obtained, the adsorption isotherms were constructed. The hyperbolic forms of Langmuir and Freundlich equations are summarized by the following expressions.

Quantity of adsorbed phosphorus:

(1) $$Q_{e}=\;\frac{\left(C_{o}-C_{e}\right)\times V}{W}$$

Quantity of desorbed phosphorus:

(2) $$D_{e}=\;\frac{C_{e}\times V}{W}$$

Langmuir adsorption isotherm curve:

(3) $$\frac{C_{e}}{Q_{e}}=\;\frac{C_{e}}{Q_{m}}+\;\frac{1}{K_{1}\times Q_{m}}$$

Maximum buffer capacity (MBC):

(4) $$\mathrm{MBC}=\;K_{1}\times Q_{m}$$

Freundlich adsorption isotherm curve:

(5) $$\mathrm{lg}Q_{e}=\mathrm{lg}K_{2}+\frac{1}{n}\mathrm{lg}C_{e}$$

where, *C_o_* (mg/L) and *C_e_* (mg/L) represent phosphorus concentration in initial and equilibrium liquid, respectively. *Q_e_* (mg/kg) and *D_e_* (mg/kg) indicate the quality of phosphorus adsorbed and desorbed per unit mass of adsorbent, respectively. *Q_m_* (maximum adsorption capacity of phosphorus, mg/kg) and MBC (maximum buffer capacity of phosphorus, mg/kg) were calculated from the slope and intercept of the regression equation performed between *C_e_* and *C_e_/Q_e_*. *K*_1_, *K*_2_ and *n* express constants associated with adsorption binding energy. *W* (g) and *V* (mL) are the adsorbent mass and the extract volume, respectively.

### 2.4. Phosphorus Sequential Fractionation

The sequential extraction scheme of Hedley et al. [26] with slightly modifications by Ruttenberg [27] was conducted for P-fractionations. Soil aggregate fractions were subjected to sequential chemical extraction with 0.5 M NaHCO_3_, 0.5 M NaOH and 1.0 M HCl. The extracts were centrifuged (8000 r/min, 12 min), and the supernatants were divided into two sub-samples. One was directly passed through 0.45-µm microfiltration membrane for determination of dissolved IP to obtain L-Pi, Fe.Al-P and Ca.Mg-P, and the other was digested with concentrated H_2_SO_4_ and K_2_S_2_O_8_ for determination of total phosphorus (WTP) to obtain L-Po (L-Po = NaHCO_3_-WTP − NaHCO_3_-Pi), Hu-P (Hu-P = NaOH-WTP −NaOH-Pi) and Ml-Po (Ml-Po = HCl-WTP −HCl-Pi). Residual phosphorus (Re-P) was evaluated from the residual digested with concentrated H_2_SO_4_ and HClO_4_.

The phosphorus recoveries of operational procedure were determined by calculating phosphorus content using two approaches. Firstly, the total amount of phosphorus (TP) in soil aggregate fractions was determined by the antimony-molybdenum colorimetric method with digestion of concentrated H_2_SO_4_ and HClO_4_. Secondly, additive phosphorus (Sum-TP) was the summation of P-fractions (Sum-TP = L-Pi + L-Po + Ml-Po + Fe.Al-P + Ca.Mg-P + Hu-P + Re-P). The comparison between TP and Sum-TP showed a reliable agreement that ranged from 86% to 117%.

### 2.5. Chemical Determination

Air-dried soil samples were analyzed for pH and EC in a 1:10 (*w/v*) sediment-to-distilled water suspension with a multi-parameter probe (520M-01A, LTD). Total nitrogen (TN) was determined through a semi-micro Kjeldahl method [5]. Fe and Mn were measured through inductively coupled plasma mass spectrometry (ICP-MS) after digestion with HNO_3_, HF and HClO_4_ [15]. The potassium dichromate heating method was used for the evaluation of SOM content [22]. Triplicate samples were analyzed, and data were expressed as mean with their standard deviation.

### 2.6. Statistical Analysis

The experimental data were calculated using Excel software Ver. 2007 (Microsoft, Redmond, WA, USA). One-way analysis of variance ANOVA and Pearson’s correlation analysis were performed using SPSS Ver. 20.0 (SPSS, Chicago, IL, USA). All figures in this study were generated with the Origin Ver. 9.0 (Microcal, Malvern, England).

## 3. Results and Analysis

### 3.1. Soil Aggregates

**Figure 1:**
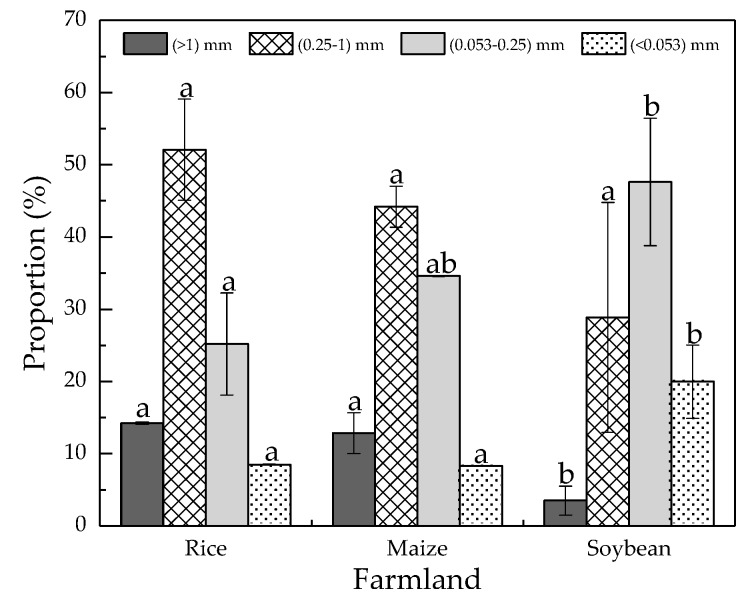
Variation in soil aggregate fractions of farmland. Note: the letters a, b indicate the significant difference in the amount of the same grain-size soil aggregate under different land uses.

A significant difference in the proportion of soil aggregates was found under different cultivated crops (Figure 1). Overall, soil aggregates were mainly S-mac and MIC, followed by L-mac and SC, and they accounted for 52.16%, 25.20%, 14.23% and 8.49% in the rice field, 44.21%, 34.61%, 12.88% and 8.30% in the maize field and 28.87%, 47.63%, 3.52% and 19.99% in the soybean field. L-mac and S-mac were in the decreasing order of rice > maize > soybean, while MIC and SC were in the increasing order of rice < maize < soybean.

**Figure 2:**
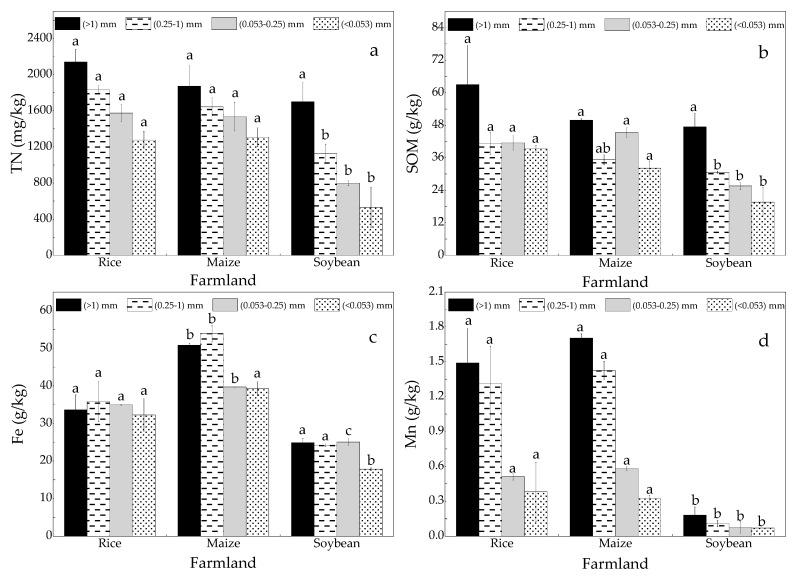
The content of (**a**) TN (Total nitrogen), (**b**) SOM (soil organic matter), (**c**) Fe and (**d**) Mn in soil aggregate fractions. Note: the letters a, b indicate the significant difference in the amount of chemical indicators in the same grain-size soil aggregate under different land uses.

TN, SOM, Fe and Mn in soil aggregate fractions decreased with the reduction in soil aggregate grain-sizes, and a significant difference was observed for different cultivated crops (Figure 2). TN and SOM in soil aggregates with different grain-sizes was in the increasing order of rice > maize > soybean. By contrast, Fe and Mn were in the increasing order of maize > rice > soybean. Mn in soybean field was significantly lower than that in rice and maize fields.

### 3.2. Phosphorus Fractions

**Figure 3:**
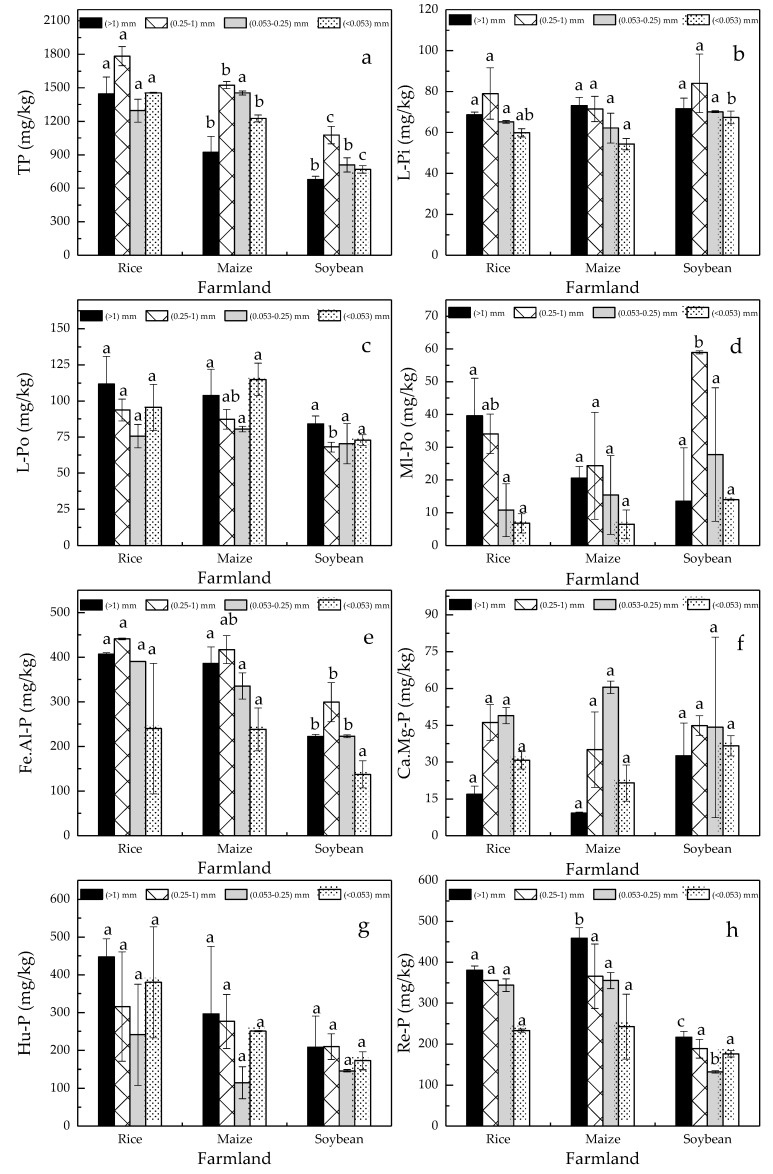
The content of (**a**) TP (total phosphorus), (**b**) L-Pi (labile inorganic phosphorus), (**c**) L-Po (labile organic phosphorus), (**d**) Ml-Po (moderately labile organic phosphorus), (**e**) Fe.Al-P (iron-aluminum bound phosphorus), (**f**) Ca.Mg-P (calcium-magnesium bound phosphorus), (**g**) Hu-P (humic phosphorus) and (**h**) Re-P (residual phosphorus) in soil aggregate fractions. Note: the letters a, b indicate the significant difference in the amount of phosphorus fractions in the same grain-size soil aggregate under different land uses.

P-fractions presented a significant difference in concentration under different cultivated crops and enrichment characteristic in soil aggregates with dissimilar grain-sizes (Figure 3). The concentration of TP in soil aggregate fractions was ranked as rice > maize > soybean, but their enrichment characteristic in soil aggregates was unconspicuous (Figure 3a). L-Pi and L-Po was approximately equivalent in soil aggregate fractions (Figure 3b,c). Fe.Al-P and Re-P tended to condense in coarse-grained aggregates (Figure 3e,h). S-mac and MIC were the primary carriers of Ca.Mg-P (Figure 3f). Cultivated crops had a prominent influence on distribution of Ml-Po and Hu-P. Specifically, the amount of Ml-Po decreased with the reduction in soil aggregate grain-size in the rice and maize fields, whereas that was concentrated in S-mac and MIC in soybean field (Figure 3d). In the rice and maize fields, Hu-P in L-mac, S-mac and SC was higher than that in MIC, whereas that was fair for soil aggregate fractions in the soybean field (Figure 3g). As for P-fraction concentration, no significant difference in L-Pi, L-Po and Ca.Mg-P was found in the rice, maize and soybean fields. Hu-P was ranked as rice > maize >soybean. Ml-Po, Fe.Al-P and Re-P were equivalent in the rice and maize fields. In the soybean field, Ml-Po was high, whereas Fe.Al-P and Re-P were low.

### 3.3. Phosphorus Adsorption Characteristic

**Figure 4:**
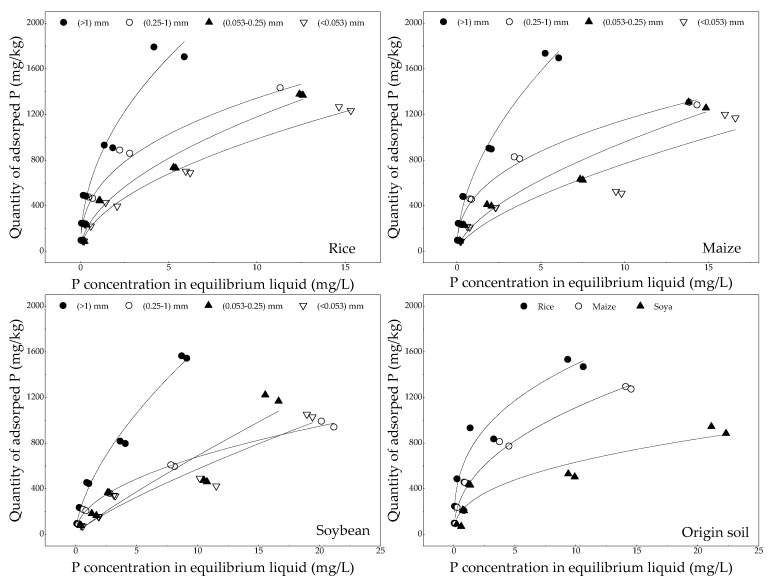
Variation in the quantity of adsorbed phosphorus with phosphorus concentration in equilibrium liquid.

The concentration of phosphorus in equilibrium liquid required for soil aggregates with different grain-sizes to reach the adsorption equilibrium was in the decreasing order of L-mac > S-mac > MIC > SC in rice, maize and soybean fields (Figure 4). This finding indicated that coarse aggregates have favorable capacity to retain phosphorus. Table 2 shows the adsorption parameters and adjustments generated for Langmuir and Freundlich models. The phosphorus adsorption in soil aggregate fractions was best fitted by the Langmuir equation (0.806 ≤ *R*^2^ ≤ 0.998) compared with Freundlich equation (0.002 ≤ *R*^2^ ≤ 0.299). Consequently, *Q_m_*, *MBC* and *K*_1_ were ranked as rice > maize > soybean and L-mac > S-mac > MIC > SC. This result implied that the ability of rice field to retain exogenous phosphorus was considerable, followed by maize and soybean fields. The amount of retained phosphorus also mainly depended on coarse aggregates.

## 4. Discussion

### 4.1. Identification of Environmental Controlling Factors of Phosphorus Fractions Distribution

Pearson’s correlation analysis results (Table 3) indicate no significant correlation between P-fractions, including L-Pi, Ca.Mg-P and L-Po, and soil chemical properties, including TN, SOM, Fe and Mn. Fe.Al-P and Re-P, have a positive correlation with TN, SOM, Fe and Mn at *p* = 0.01. L-Po is positively correlated with TN and SOM at *p* = 0.01, and it is positively correlated with Fe and Mn at *p* = 0.05. A positive correlation (*p* = 0.05) with TN, SOM and Mn was observed for Hu-P and TN, Fe and Mn for TP.

Nitrogen and phosphorus are primary limiting elements for wetland ecosystems. The ratio of nitrogen to phosphorus (N:P) has an indirect influence on the distribution and transformation of P-fractions by effecting vegetation communities and densities and microbial species and activity [16,28]. Fan et al. [28] indicated that nitrogen and phosphorus collectively restrict the incubation process of terrestrial ecosystems, including wetland, agricultural, forest and pasture organisms, and their interaction stimulates a unified complex formation of ecosystems. Previous research has demonstrated that N-enriched soil can promote the conversion of Hu-P to labile phosphorus, including L-Pi, L-Po and Ml-Po [29,30,31]. George et al. [32] suggested that nitrogen provides essential nutrients for microorganisms, and the organic phosphorus (OP) attached to adsorbed substrate can be released under the mineralization of microorganisms. When N:P ≤ 25, the mineralized decomposition of SOM will accelerate the release of phosphorus combined with organic matter with metal cations as a binder [33].

SOM, which is a cementing agent for OP, dominates the bio-geochemical circulation of P-fractions by affecting the grain size and water stability of soil aggregates and microbial community structure [34,35]. Gu et al. [36] also demonstrated that released phosphorus can come from dissolved microbial cells and phosphorus-laden micro-aggregates. However, SOM has a double-sided influence on soil phosphorus retention-release. SOM can generally form an organic complex with soil minerals under the action of iron-aluminum and calcium-magnesium bonds, thereby improving the soil capacity to retain phosphorus [10]. However, the organic anions decomposed with SOM mineralization compete with phosphate for the adsorption sites distributed on metal oxides [16]. In addition, elevated SOM leads to a lower fixation degree of phosphorus to soil particles; this condition increases the mobility of phosphorus with surface flowing water [23].

Numerous studies have demonstrated that metal oxides and their hydroxides can provide abundant adsorption sites for phosphorus; thus, elevated metal content can increase soil retention capacity to endogenous and exogenous phosphorus [11,37]. However, this mechanism is considerably affected by pH and oxidation-reduction potential (ORP). In the meantime, pH influences the precipitation and dissolution of soil Fe and Al oxides because acidification transforms crystalline Fe and Al oxides into water-soluble Fe and Al and then produces amorphous Fe-Al hydroxides by hydrolysis [28]. Under acidic conditions, carbon dioxide produced by microbial metabolism can promote the dissolution of Ca.Mg-P [6], whereas H^+^ can protonate the groups on the surface of the iron-aluminum oxide; ultimately, the amount of phosphorus adsorbed by soil increases [37]. Under alkaline conditions, OH^−^ replaces the phosphate adsorbed on the surface of iron-aluminum oxidation as a function of ion exchange, thereby resulting in the release of phosphate [9]. Under aerobic conditions, metal ions, such as iron and manganese, are generally oxidized to their higher states (Fe^3+^ and Mn^4+^) and will precipitate phosphorus with metal hydroxide; by contrast, the iron and manganese precipitates will dissolve and release phosphate under reduction conditions [38]. The aforementioned conclusions indicate Fe and Mn oxides are the dominating minerals that control phosphorus solubility [28,39], but phosphorus saturation status will be destroyed when pH and ORP change.

### 4.2. Phosphorus Availability and Release under Different Cultivated Crops

Easily-available phosphorus, including L-Pi, L-Po and Ml-Po, is an indicator of time-sensitive bioavailability of phosphorus; and moderately-available phosphorus, including Fe.Al-P and Hu-P, represents the short-term bioavailability, whereas non-available phosphorus, including Ca.Mg-P and Re-P, represents the long-term bioavailability [40,41]. In Sanjiang Plain, the contents of TP in maize, soybean and rice fields were 1052.54, 893.60 and 792.46 mg/kg, respectively. With regard to easily-available phosphorus, the sums of L-Pi, L-Po and Ml-Po are 136.40, 104.22 and 81.20 mg/kg, accounting for 17.21%, 11.66% and 7.71% of TP in rice, maize and soybean fields, respectively. This conclusion demonstrates plant-available phosphorus in rice fields is higher than in maize and soybean fields. The satisfactory P-availability in rice fields can also be attributed to considerable TN and SOM amounts (Table 1). The reason is that TN and SOM can provide abundant nutrients and carbon sources for the growth of microorganisms and indirectly promote the mineralization and decomposition of OP (Hu-P) [42,43]. Fe.Al-P and Hu-P are considered to be moderately-available phosphorus that involves long-term soil phosphorus transformation and act as a buffer for easily-available phosphorus in agro-ecosystems. The sums of Fe.Al-P and Hu-P in rice and soybean fields are equivalent, which are 388.44 and 368.82 mg/kg, respectively, whereas that in maize fields is higher with a value of 633.19 mg/kg. The aforementioned results indicate that maize fields have a considerable potential for phosphorus supply under variation in environmental conditions, such as a decrease in oxidation reduction potential (ORP) and an increase in microbial activity [16,44]. Fe.Al-P, which is a redox-sensitive P-fraction, is released as Fe^3+^ is reduced to Fe^2+^ under anaerobic conditions [45]. In the meantime, the anaerobic conditions can also destroy the metal bridge (such as Fe^3+^ and Al^3+^) that binds SOM and phosphorus [10], thereby leading to the release of Hu-P. Therefore, a flooding environment is conducive to converting moderately-available phosphorus to easily-available phosphorus in rice fields. In maize fields, high levels of Fe and Mn (41.92 and 1.27 g/kg) are responsible for the large content of Fe.Al-P and the potential for phosphorus supply. Under aerobic conditions, the oxides and hydroxides of Fe and Mn provide abundant adsorption sites for phosphorus and increase amount of retained phosphorus [46]. Ca.Mg-P, which is derived from the insoluble calcium phosphate mineral [7,38], and Re-P, which has strong binning energy in the mineral lattice [47], are characterized as nonvalent P-fractions and scarcely utilized as a nutrient by plants. In rice fields, the sum of Ca.Mg-P and Re-P is only 147.07 mg/kg, accounting for 18.56% of TP, whereas the sums in maize and soybean fields are 311.11 and 280.08 mg/kg, accounting for 29.56% and 31.34% of TP, respectively. This finding is consistent with the high time-sensitive bioavailability of phosphorus in rice fields.

Adsorption isotherm simulation results show that coarse aggregates have high capacity to retain phosphorus (Figure 4). In the meantime, fine-grained aggregates easily migrate with surface runoff. The contents of coarse aggregates (L-mac and S-mac) are ranked as rice > maize > soybean, whereas the contents of fine aggregates (MIC and SC) are ranked as rice < maize < soybean (Figure 2). Therefore, from the perspective of soil aggregates, the release risk of phosphorus in rice fields is lower than those in maize and soybean fields. Labile phosphorus, including L-Pi, L-Po and Ml-Po, has low content and high plant utilization, so it makes little contribution to agricultural NPS phosphorus pollution. Fe.Al-P and Hu-P are higher in soil aggregates with different grain-sizes in rice fields than those in maize and soybean fields. Unfortunately, the flooded environment of rice fields will promote their release. Therefore, the drainage period of rice fields should be strictly controlled. Acidic conditions in farmland (5.29, 5.21 and 5.34) (Table 1) facilitate the release of Ca.Mg-P because H^+^ can aggravate its dissolution [37,38]. Re-P is nonvalent with a low possibility of release through morphological transformation [17]. However, the migration of phosphorus concentrated in soil aggregates with runoff should be focused. In summary, P-fractions in rice fields easily change under aerobic–anaerobic conditions. Therefore, the risk of phosphorus loss during drainage should be given considerable attention.

## 5. Conclusions

Different cultivated crops significantly influenced the grain-size of soil aggregates and the distribution of P-fractions. In agro-ecosystems, soil aggregates were mainly S-mac and MIC, followed by L-mac and SC, accounting for 52.16%, 25.20%, 14.23% and 8.49% in the rice field, 44.21%, 34.61%, 12.88% and 8.30% in the maize field, and 28.87%, 47.63%, 3.52% and 19.99% in the soybean field. The concentrations of TN, SOM, Fe and Mn in soil aggregate fractions decreased with the reduction in soil aggregate grain-sizes. Fe.Al-P and Re-P tended to condense in L-mac and S-mac. MIC and SC were the primary carriers of Ca.Mg-P. Coarse aggregates had strong capacity to retain phosphorus. In rice fields, phosphorus bioavailability and utilization rate were high. However, the P-fractions in there are easily changed with aerobic–anaerobic conditions. Therefore, attention should be paid to the risk of phosphorus loss during drainage.

## Figures and Tables

**Table 1:** Chemical properties of soil samples under different land uses.

Farmland	pH	EC	TN	TP	SOM	Fe	Mn
	-	µs/cm	mg/kg	mg/kg	g/kg	g/kg	g/kg
Rice	5.29 ± 0.21 ^a^	82.45 ± 9.98 ^a^	1802.75 ± 176.42 ^a^	792.46 ± 175.97 ^a^	47.55 ± 9.83 ^a^	35.96 ± 1.02 ^a^	1.34 ± 0.40 ^a^
Maize	5.21 ± 0.04 ^a^	57.29 ± 6.25 ^a^	1715.50 ± 94.05 ^a^	1052.54 ± 341.70 ^a^	44.15 ± 1.34 ^a^	41.92 ± 3.85 ^a^	1.27 ± 0.13 ^a^
Soybean	5.34 ± 0.01 ^a^	78.89 ± 6.93 ^a^	1016.25 ± 238.6 ^b^	893.60 ± 24.91 ^a^	28.05 ± 5.30 ^a^	20.34 ± 1.44 ^b^	0.22 ± 0.02 ^b^

Note: the letters ^a^, ^b^ indicate the significant difference in the chemical properties of soil samples under different land uses. TN: Total nitrogen; TP: total phosphorus; SOM: soil organic matter.

**Table 2:** The results of adsorption isotherm simulation.

Soil Aggregate Fractions	Farmland	Langmuir [*C_e_*/*Q_e_* = *C_e_*/*Q_m_* + 1/(*K*_1_·*Q_m_*)]				Freundlich [lg*Q_e_* = lg*K*_2_ + 1/n·lg*C_e_*]
		*Q_m_*	MBC	*K*_1_	*R* ^2^	*R* ^2^
**mm**	**-**	**mg/kg**	**mg/kg**	**-**	**-**	**-**
>1	Rice	2020.41 ± 28.86 ^a^	2239.82 ± 991.87 ^a^	1.11 ± 0.51 ^a^	0.930	0.266
	Maize	2037.04 ± 261.89 ^a^	1605.39 ± 502.60 ^a^	0.81 ± 0.35 ^a^	0.917	0.276
	Soybean	1923.08 ± 0.00 ^a^	654.01 ± 78.08 ^a^	0.34 ± 0.04 ^a^	0.876	0.008
0.25–1	Rice	1562.50 ± 0.00 ^a^	1204.98 ± 406.66 ^a^	0.77 ± 0.26 ^a^	0.985	0.103
	Maize	1440.71 ± 73.29 ^a^	872.95 ± 238.61 ^a^	0.61 ± 0.20 ^a^	0.982	0.002
	Soybean	1120.15 ± 79.65 ^b^	266.42 ± 28.45 ^a^	0.24 ± 0.04 ^a^	0.949	0.135
0.053–0.25	Rice	1590.91 ± 107.14 ^a^	520.07 ± 101.32 ^a^	0.33 ± 0.09 ^a^	0.888	0.044
	Maize	1399.22 ± 41.51 ^a^	357.13 ± 36.78 ^a^	0.26 ± 0.03 ^a^	0.806	0.299
	Soybean	594.23 ± 54.70 ^b^	229.31 ± 58.96 ^a^	0.39 ± 0.14 ^a^	0.960	0.057
<0.053	Rice	1459.93 ± 15.07 ^a^	395.60 ± 29.79 ^a^	0.25 ± 0.02 ^a^	0.903	0.224
	Maize	583.13 ± 7.21 ^b^	487.37 ± 55.07 ^a^	0.21 ± 0.02 ^a^	0.998	0.201
	Soybean	676.45 ± 183.85 ^b^	149.06 ± 28.09 ^b^	0.68 ± 0.13 ^b^	0.900	0.277

Note: the letters ^a^, ^b^ indicate the significant difference in simulation parameters of the same grain-size aggregate under different land uses. MBC: Maximum buffer capacity.

**Table 3:** Correlation analysis results between phosphorus fractions and soil chemical properties.

Chemical Properties	L-Pi	L-Po	Ml-Po	Fe.Al-P	Ca.Mg-P	Hu-P	Re-P	TP
TN	0.125	0.577 **	0.109	0.766 **	−0.292	0.405 *	0.786 **	0.497 *
SOM	−0.019	0.547 **	0.123	0.566 **	−0.314	0.499 *	0.655 **	0.339
Fe	−0.072	0.451 *	−0.132	0.626 **	−0.261	0.231	0.771 **	0.484 *
Mn	0.212	0.457 *	0.138	0.742 **	−0.371	0.463 **	0.862 **	0.486 *

* Correlation is significant at the 0.05 level; ** Correlation is significant at the 0.01 level.
